# Underrepresented Populations in Parkinson's Genetics Research: Current Landscape and Future Directions

**DOI:** 10.1002/mds.29126

**Published:** 2022-07-22

**Authors:** Artur Francisco Schumacher‐Schuh, Andrei Bieger, Olaitan Okunoye, Kin Ying Mok, Shen‐Yang Lim, Soraya Bardien, Azlina Ahmad‐Annuar, Bruno Lopes Santos‐Lobato, Matheus Zschornack Strelow, Mohamed Salama, Shilpa C. Rao, Yared Zenebe Zewde, Saiesha Dindayal, Jihan Azar, Lingappa Kukkle Prashanth, Roopa Rajan, Alastair J. Noyce, Njideka Okubadejo, Mie Rizig, Suzanne Lesage, Ignacio Fernandez Mata, Global Parkinson's Genetics Program (GP2), Emilia Gatto, Marcelo Kauffman, Julie Hunter, Kishore Kumar, Miguel E. Renteria, Sulev Koks, Alexander Zimprich, Artur Francisco Schumacher Schuh, Bruno Santos, Carlos Rieder, Vitor Tumas, Oury Monchi, Ted Fon, Marcelo David Miranda, Maria Leonor Bustamante, Patricio Olguin, Pedro Chana, Beisha Tang, Hui‐Fang Shang, Ji Feng Guo, Piu Chan, Wei Luo, Gonzalo Arboleda, Jorge Luis Orozco, Marlene Jimenez del Rio, Alvaro Hernandez, Mohamed Salama, Biniyam Ayele, Yared Zenebe, Alexis Brice, Jean‐Christophe Corvol, Anastasia Illarionova, Brit Mollenhauer, Christine Klein, Eva‐Juliane Vollstedt, Katja Lohmann, Lara Mariah Lange, Manu Sharma, Peter Heutink, Thomas Gasser, Zih‐Hua Fang, Albert Akpalu, Georgia Xiromerisiou, Leonidas Stefanis, Andrew Sobering, Alex Medina, Germaine Chan, Nancy Ip, Nelson Yuk‐Fai Cheung, Phillip Chan, XiaoPu Zhou, Asha Kishore, Pramod Pal, Roopa Rajan, Rupam Borgohain, Enza Maria Valente, Micol Avenali, Tommaso Schirinzi, Manabu Funayama, Nobu Hattori, Tomotaka Shiraishi, Tomotaka Shiraishi, Rejko Kruger, Ai Huey Tan, Azlina Ahmad‐Annuar, Nor Azian Abdul Murad, Norlinah Mohamed Ibrahim, Shahrul Azmin, Shen‐Yang Lim, Wael Mohamed, Daniel Martinez, Mayela Rodríguez Violante, Tim Anderson, Toni Pitcher, Njideka Okubadejo, Oluwadamilola Ojo, Jan Aasly, Lasse Pihlstrøm, Shoaib Urrehman, Mario Cornejo‐Olivas, Angel Vinuela, Elena Iakovenko, Jia Nee Foo, Soraya Bardien, Yun Joong Kim, Alejandro Martinez Carrasco, Janet Hoenicka, Sarah Elsadig, Chin‐Shien Lin, Ruey‐Meei Robin Wu, Serena Wu, Yih‐Ru Wu, Samia Ben Sassi, Gencer Genc, Nazli Basak, Özgür Öztop Çakmak, Sibel Ertan, Alastair Noyce, Camille Carroll, Claire Bale, Clodagh Towns, Henry Houlden, Huw Morris, John Hardy, Joseph Callanan, Kin Mok, Manuela Tan, Mie Rizig, Nick Wood, Nigel Williams, Olaitan Okunoye, Patrick Lewis, Rauan Kaiyrzanov, Rimona Weil, Simon Stott, Sumit Dey, Alyssa O'Grady, Bradford Casey, Caroline Pantazis, Claire Wegel, Dan Vitale, Deborah Hall, Ejaz Shamim, Faraz Faghri, Hampton Leonard, Hirotaka Iwaki, Ignacio Fernandez Mata, Ignacio Juan Keller Sarmiento, Jeff Kim, Joshua Shulman, Justin C. Solle, Karl Kieburtz, Ken Marek, Lana Chahine, Laurel Screven, Lisa Shulman, Maggie Kuhl, Marissa Dean, Mary Makarious, Miguel Inca, Mike Nalls, Niccolo Mencacci, Roy Alcalay, Ruqaya Murtadha, Sara Bandres‐Ciga, Schuyler Fox, Sohini Chowdhury, Steven Lubbe, Tao Xie, Tatiana Foroud, Todd Sherer, Yeajin Song, Andrew Singleton, Bernadette Siddiqi, Brian Fiske, Cornelis Blauwendraat, Ekemini Riley, Duan Nguyen, Toan Nguyen, Masharip Atadzhanov

**Affiliations:** ^1^ Departamento de Farmacologia Universidade Federal do Rio Grande do Sul Porto Alegre Brazil; ^2^ Serviço de Neurologia Hospital de Clínicas de Porto Alegre Porto Alegre RS Brazil; ^3^ Graduate Program in Biological Sciences: Biochemistry Universidade Federal do Rio Grande do Sul Porto Alegre Brazil; ^4^ Department of Clinical and Movement Neurosciences Queen Square Institute of Neurology, University College London United Kingdom; ^5^ Department of Neurodegenerative Disease and UK Dementia Research Institute University College of London London United Kingdom; ^6^ Division of Life Sciences Hong Kong University of Science and Technology Hong Kong China; ^7^ Division of Neurology, Department of Medicine, and the Mah Pooi Soo & Tan Chin Nam Centre for Parkinson's & Related Disorders, Faculty of Medicine University of Malaya Kuala Lumpur Malaysia; ^8^ Division of Molecular Biology and Human Genetics, Department of Biomedical Sciences, Faculty of Medicine and Health Sciences Stellenbosch University Cape Town South Africa; ^9^ Department of Biomedical Science, Faculty of Medicine University of Malaya Kuala Lumpur Malaysia; ^10^ Laboratório de Neuropatologia Experimental Universidade Federal do Pará Belém Brazil; ^11^ Graduate Program in Medicine: Medical Sciences Universidade Federal do Rio Grande do Sul Porto Alegre Brazil; ^12^ Institute of Global Health and Human Ecology The American University in Cairo Cairo Egypt; ^13^ Genomic Medicine Institute, Lerner Research Institute, Genomic Medicine Cleveland Clinic Foundation Cleveland Ohio USA; ^14^ Department of Neurology College of Health Sciences, Addis Ababa University Addis Ababa Ethiopia; ^15^ Division of Neurology, Department of Neurosciences, Faculty of Health Sciences University of the Witwatersrand Johannesburg South Africa; ^16^ Center for Parkinson's Disease and Movement Disorders Manipal Hospital Bangalore India; ^17^ Department of Neurology All India Institute of Medical Sciences (AIIMS) New Delhi India; ^18^ Preventive Neurology Unit, Centre for Prevention, Detection and Diagnosis Wolfson Institute of Population Health, Queen Mary University of London London United Kingdom; ^19^ Department of Medicine College of Medicine, University of Lagos Lagos State Nigeria; ^20^ Institute of Neurology, University College of London London United Kingdom; ^21^ Sorbonne Université, Institut du Cerveau—Paris Brain Institute—ICM, INSERM, CNRS, Assistance Publique Hôpitaux de Paris, Hôpital Pitié‐Salpêtrière, CIC Neurosciences Paris France

**Keywords:** Parkinson's disease, systematic review, diversity, underrepresented populations, genetics

## Abstract

**Background:**

Human genetics research lacks diversity; over 80% of genome‐wide association studies have been conducted on individuals of European ancestry. In addition to limiting insights regarding disease mechanisms, disproportionate representation can create disparities preventing equitable implementation of personalized medicine.

**Objective:**

This systematic review provides an overview of research involving Parkinson's disease (PD) genetics in underrepresented populations (URP) and sets a baseline to measure the future impact of current efforts in those populations.

**Methods:**

We searched PubMed and EMBASE until October 2021 using search strings for “PD,” “genetics,” the main “URP,” and and the countries in Latin America, Caribbean, Africa, Asia, and Oceania (excluding Australia and New Zealand). Inclusion criteria were original studies, written in English, reporting genetic results on PD from non‐European populations. Two levels of independent reviewers identified and extracted information.

**Results:**

We observed imbalances in PD genetic studies among URPs. Asian participants from Greater China were described in the majority of the articles published (57%), but other populations were less well studied; for example, Blacks were represented in just 4.0% of the publications. Also, although idiopathic PD was more studied than monogenic forms of the disease, most studies analyzed a limited number of genetic variants. We identified just nine studies using a genome‐wide approach published up to 2021, including URPs.

**Conclusion:**

This review provides insight into the significant lack of population diversity in PD research highlighting the immediate need for better representation. The Global Parkinson's Genetics Program (GP2) and similar initiatives aim to impact research in URPs, and the early metrics presented here can be used to measure progress in the field of PD genetics in the future. © 2022 The Authors. *Movement Disorders* published by Wiley Periodicals LLC on behalf of International Parkinson and Movement Disorder Society.

Since the Human Genome Project, the development of new technologies for the interrogation of genetic variability has increased exponentially, and new large‐scale, high‐throughput sequencing methods for genotyping and DNA sequencing have emerged, allowing large numbers of genome‐wide association studies (GWASs) to be performed. These technologies and the resulting analyses have revolutionized genetic investigation of disease; however, as pointed out by previous analyses of GWAS databases, these studies have failed in one major regard: they are not representative of the global genetic diversity. As a consequence of sample availability, budgetary constraints, issues with enrollment, or statistical power, populations of European ancestry still represent the majority of subjects included.^1^, ^2^ This lack of diversity has resulted in missed opportunities, such as the discovery of new genetic associations for complex traits and the discovery of novel genetic causes of monogenic forms of disease that could help unveil unknown causes of these pathologies. It also threatens to jeopardize medical care, drug development, and advancements in precision medicine, preventing equitable health care among different populations.^3^, ^4^, ^5^

Parkinson's disease (PD) is a multifactorial disorder in which a complex interaction between genetics and environmental factors occurs. As no curative or preventive therapy is currently available, exploring its pathophysiology is crucial to improve treatment. To date, approximately 20 genes with highly penetrant rare variants are related to familial or monogenic forms of PD, predominantly among persons of European ancestry.^6^ A recent GWAS meta‐analysis nominated 90 risk variants explaining approximately a quarter of the disease heritability. However, this study included just individuals of European ancestral origin, limiting the generalizability of these discoveries to other populations.^7^ The largest PD GWAS among non‐Europeans was recently reported in East Asians.^8^ The report included almost 7000 individuals with PD and identified two novel risk loci. Research on PD genetics has increased in the past two decades, but a lack of diversity remains a significant problem for understanding the biological basis of the disease in all populations.

Many researchers are aware of the problem elicited by the lack of inclusion of underrepresented populations (URPs) and the hazards that result from avoiding or not achieving diversity within PD genetic studies. Nevertheless, most of the publications that raised this matter comprise comments, editorials, and letters, with only a few of them relying on empirical data.^9^ Notwithstanding the value of these reports, which helped shed light on the issue, an in‐depth understanding of the geographic and ethnic coverage of PD genetic studies is necessary for building a solid roadmap for increasing diversity. This systematic review and bibliometric analysis aims to provide an overview of the publications in PD genetics in URPs (individuals of non‐European ancestry) to date, thereby clarifying the main gaps, identifying opportunities to ensure more diversity, and setting a baseline to measure the impact of future global efforts.

## Patients and Methods

We searched PubMed/MEDLINE and EMBASE from inception through October 2021. The search strings for each database were created using terms for “Parkinson's disease,” “genetics,” “main non‐European ethnic groups,” and the countries in Latin America, Caribbean, Africa, Asia, and Oceania (excluding Australia and New Zealand) (Supplementary Table 1). Inclusion criteria were original studies reporting genetic results on PD from non‐European populations and published in English. Systematic and narrative reviews, meta‐analyses, and papers reporting exclusively functional, epigenetic, or biomarker results were excluded.

Rayyan software was used to detect duplicates and perform the first screening procedure.^10^ We implemented the review in a two‐step approach. First, two independent researchers screened titles and abstracts for inclusion criteria, and a third reviewer judged any discrepancies. Second, another reviewer examined the entire content of the selected papers to reassess inclusion criteria and collect data through an online extraction form. We collected information on the study design for each included study, classified as a study of familial/monogenic cases, sporadic PD, or GWAS. Studies were defined as involving familial/monogenic forms of PD if they included subjects with an autosomal dominant or recessive family history of PD or if they reported results in known causative mutations in PD genes. For statistical purposes, if a study included both sporadic and familial/monogenic cases and this were clearly explicit in the text, the same study was included in both categories. Ethnicity was mainly categorized by geographical perspective, and we determined it based on the explicit description in the manuscript or inferred by the country of origin. Laboratory methods used for genetic analyses are very diverse, but to measure the access to technologies, we highlight those using next‐generation sequencing (considered a “new” technology). The collaborative network was defined based on the number of distinct centers collecting samples, and we classify them as single‐center and multicenter within the same country or international multicenter. Finally, funding information was classified as funded exclusively from sponsors located in underrepresented regions or not.

Bibliometric analysis was also conducted based on the titles previously selected that had a full record in the Web of Science Core Collection database. From this database, we retrieved the number of authors and citations per document, the impact factor of the journals, studies with authors from single or multiple countries, and the collaborative network among authors from different countries. Graph theory measures were retrieved at network and country levels and directly compared. For comparative analysis of the quality and visibility of the studies published, we reran the PubMed search without applying any exclusion criteria for the most productive countries in underrepresented regions. We compared the results with the same search for three different countries with mainly European ancestry from different continents (Germany, Canada, and Australia). Descriptive and comparative analyses between main ethnicities were performed in Python 3.9.5 and R 4.0.5, using the package “bibliometrix.”^11^ Raw and derived data supporting the findings of this study are available from the corresponding author on request.

## Results

After the duplicates were removed, we retrieved 2606 titles from the search in PubMed/MEDLINE and EMBASE, from which 1312 were excluded in the first screening step, resulting in 1294 papers (Supplementary Figure 1). In the second step, when the entire paper was examined, 255 papers were excluded, resulting in a final count of 1037 (for a completed list of references included, see Supplementary Table 2). The main reasons for exclusion were as follows: studies were not written in English (n = 88)—74 in Chinese, 7 in Spanish, 6 in Japanese, and 1 in Persian; none of them have performed a GWAS when examining their titles and abstracts. Other reasons for exclusions were as follows: individuals from URPs were not included, persons with PD were not included, no genetic analysis was performed, and original results were not reported. For a subset of papers (n = 997) that were also available in the Web of Science Core Collection database, further bibliometric analysis was performed.

**FIG 1:**
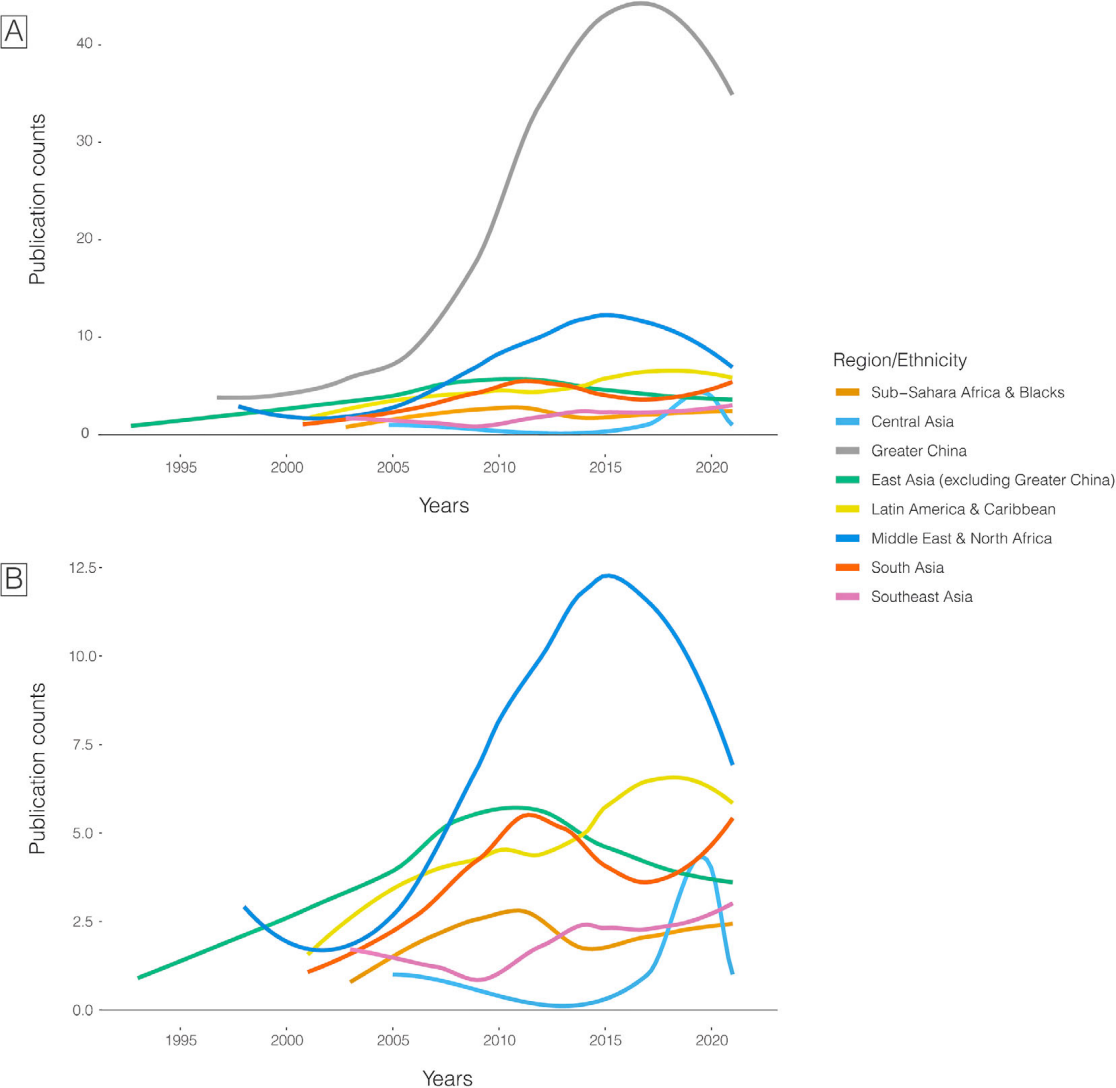
Publication counts by region/ethnicity along the years. Panel A: all regions/ethnicities included. Panel B: excluding Greater China to see the pattern of other regions/ethnicities more clearly.

The first paper retrieved in our search was published in 1993, and we observed a trend of increasing publication counts each year, with 98 published in 2020. However, the only consistent increase in publication counts along the years was among the Greater China region, whereas those among persons from Central and Southeast Asia and sub‐Saharan Africa or Black ancestry showed the lowest increases (Fig. 1). Interestingly, we observe a decrease in publication counts for Latin America and Caribbean and the Middle East and North Africa in the previous 5 years. Overall, PD genetic publications from URPs were dominated by participants from Greater China (n = 589, 57%), followed by participants from the Middle East and North Africa (n = 172, 17%), East Asia excluding Greater China (n = 106, 10%), Latin America and Caribbean (n = 102, 10%), South Asia (n = 80, 8%), sub‐Saharan Africa or other Blacks (n = 37, 4%), and Southeast Asia (n = 35, 3%). Just five publications were identified describing research from Central Asia.

**FIG 2:**
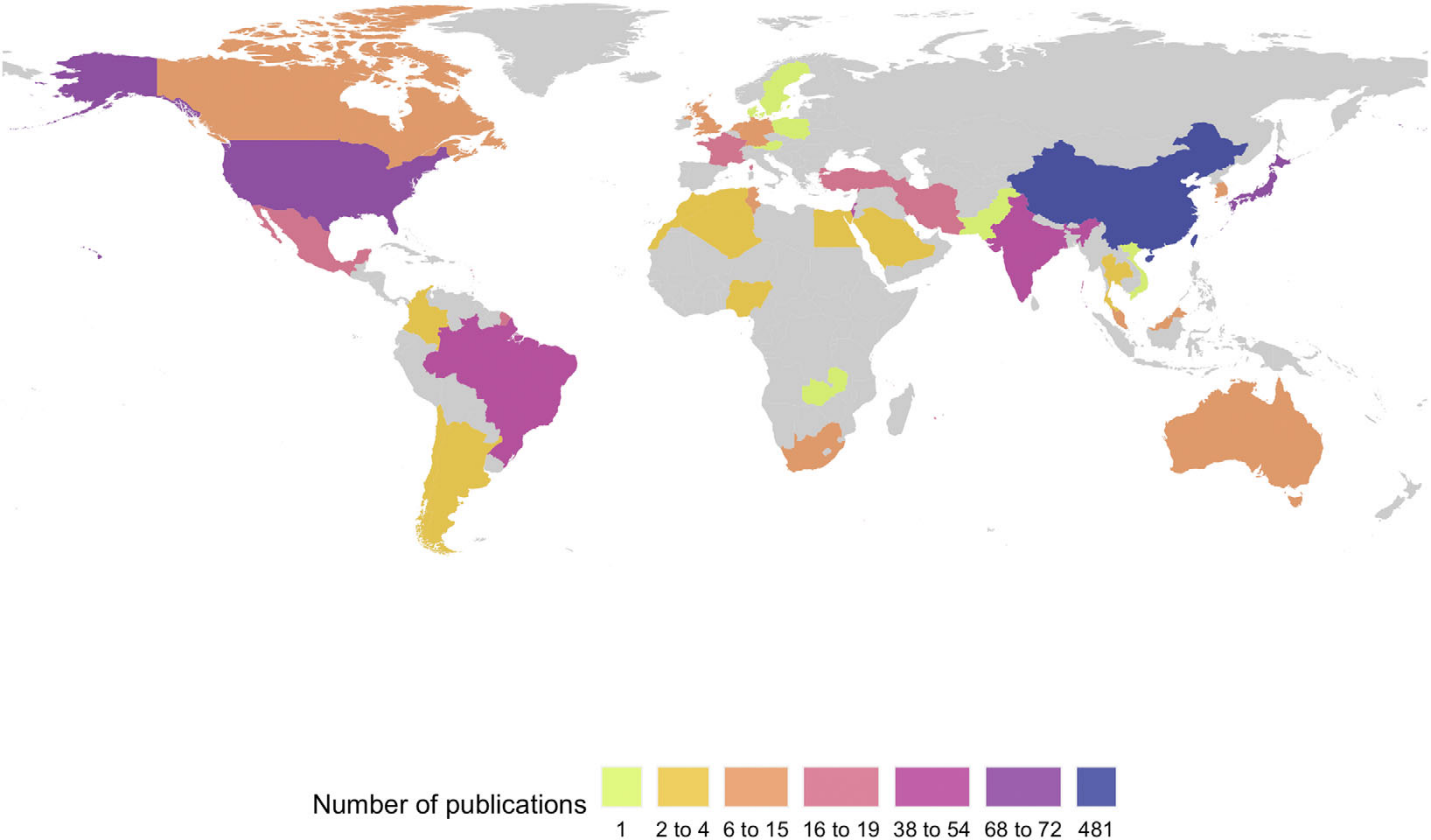
Publication counts by corresponding author's countries/regions.

Considering the corresponding author's countries/regions, which were not necessarily synonymous with the ethnic groups studied, the Greater China region has the highest number of papers (481, 48.3%), followed by Japan (72, 7.2%), the United States (68, 6.8%), India (54, 5.4%), and Brazil (44, 4.2%) (Fig. 2). Studies including participants of Chinese ancestry were mainly from the Greater China region (480, 84.9%) and Singapore (34, 6.0%). East Asia excluding Greater China were mainly from Japan (71, 67.6%) and South Korea (15, 14.3%); South Asia from India (54, 70%) and South Africa (9, 11.5%); Southeast Asia from Singapore (11, 34.4%), Malaysia (8, 25%), and Japan and Thailand (4, 12.5% each); the Middle East and North Africa from Israel (41, 25.5%), the United States (27, 16.8%), Iran (19, 11.8%), and France and Turkey (14, 8.7%, each); Latin America and Caribbean from Brazil (44, 42.7%), the United States (19, 18.5%), and Mexico (16, 15.5%); and sub‐Saharan Africa or other Blacks from South Africa (15, 36.8%), the United States (11, 30%), France (4, 10.5%), and Nigeria (3, 8%). All papers from Central Asia were from the corresponding author's countries outside the region.

Most of the scientific production analyzed concentrated on case–control studies of genetic risk factors or genotype–phenotype associations of sporadic PD (Table 1). Monogenic/familial forms of PD were the focus of 351 (33.9%) studies, with 75.3% of them reporting results on a single gene, mostly by conventional Sanger sequencing methods (62.2%), and the use of whole‐exome sequencing was mostly observed among Chinese (18.9%, Supplementary Table 3). Greater China studies tended to focus on idiopathic PD, with less emphasis on monogenic/familial forms of PD (20.7%). In contrast, this latter type of study was more represented in the other groups, especially in sub‐Saharan Africa or other Blacks (78.4%), Central Asia (60.0%), and Middle East and North Africa (55.2%). GWASs were described only in nine (0.9%) papers (Supplementary Table 4); one of the most recent was published in 2020 and recruited almost 7000 Asian patients for the discovery sample, being the largest cohort of PD patients among URP studies to date.^8^ Recently, a GWAS study among Latinos was published with almost 1500 participants.^12^ The use of next‐generation targeted sequencing, whole‐exome, and whole‐genome sequencing was present in only 9.3% of studies, mostly in East Asians non‐Chinese (11.3%) and in Chinese (9.9%). Sub‐Saharan Africans and other Blacks and Latin Americans and Caribbeans presented the lowest relative use of these technologies (5.4% and 2.9%, respectively, Table 1 and Supplementary Table 5). Studies were funded exclusively by local resources in more than 70% of the studies in Greater China and South and Southeast Asia; in Latin America, this number was approximately 50%, and in Africa and the Middle East, it decreased to less than 30%. Unfortunately, we were not able to retrieve information on the nature of the funding (government vs. nonprofit organizations vs. private sector) because of the poor reporting of this information in the papers and the difficulty to obtain more detailed information from the web pages of the funding institutions.

**TABLE 1:** Characteristics of the studies according to main ethnicities/global regions

	All studies	Greater China	East Asian (other than Greater China)	South Asia	Southeast Asia	Central Asia	Latin America and Caribbean	Middle East and North Africa	Sub‐Saharan African or other Blacks
Count^a^ (%)	1037	589 (57)	106 (10)	80 (8)	35 (3)	5 (0.5)	102 (10)	172 (17)	37 (4)
Average years from publication, mean (SD)	7.6 (5.55)	7.19 (5.34)	11.14 (6.87)	7.9 (5.17)	8.37 (5.94)	3.8 (6.83)	8.31 (5.5)	7.67 (4.96)	8.84 (4.73)
Case reports and case series,^b^ count (%)	52 (5.02)	18 (3.06)	6 (5.66)	4 (5.0)	1 (2.86)	0 (0.0)	6 (5.88)	17 (9.88)	3 (8.11)
Sample size of PD patients, median, (interquartile range)	250.0 (105.0–512.25)	383.0 (178.0–583.0)	229.0 (116.75–500.25)	157.0 (90.5–292.5)	386.0 (155.0–630.0)	80.5 (53.0–141.75)	138.0 (81.5–219.25)	151.0 (59.0–422.0)	104.0 (39.0–229.0)
Sample size of controls, median, (interquartile range)	263.0 (100.0–499.75)	368.5 (146.75–549.25)	275.0 (122.0–422.0)	170.0 (100.0–305.0)	374.0 (158.0–507.0)	61.5 (55.25–67.75)	122.0 (1.0–208.0)	110.0 (32.0–378.0)	121.5 (0.0–213.75)
Authors per document, mean (SD)	9.89 (7.37)	9.22 (6.79)	13.35 (10.67)	7.08 (3.43)	13.75 (10.23)	16.86 (13.57)	13.51 (12.9)	12.73 (8.89)	9.47 (8.62)
Citations per year per document, mean (SD)	2.21 (4.35)	1.82 (4.11)	4.23 (10.96)	1.53 (1.36)	5.18 (15.13)	4.83 (9.9)	3.48 (8.91)	4.09 (7.77)	1.75 (1.23)
Journal's impact factor, mean (SD)	3.87 (3.88)	4.35 (5.23)	6.13 (8.43)	3.45 (1.97)	7.58 (13.36)	4.86 (3.79)	5.37 (9.71)	6.82 (10.09)	3.95 (2.17)
All authors from the same country, count (%)	707 (70.98)	431 (76.28)	75 (71.43)	66 (84.62)	16 (50)	2 (28.57)	65 (63.11)	62 (38.51)	19 (50)
Studies with authors from multiple countries, count (%)	289 (29.02)	134 (23.72)	30 (28.57)	12 (15.38)	16 (50)	5 (71.43)	38 (36.89)	99 (61.49)	19 (50)
Studies on monogenic/familial PD, count (%)	351.0 (33.88)	122.0 (20.71)	45.0 (42.45)	42.0 (52.5)	11.0 (31.43)	3.0 (60.0)	52.0 (50.98)	95.0 (55.23)	29.0 (78.38)
Exclusively funded by a URP country, count (%)	658 (63.51)	452 (76.74)	61 (57.55)	58 (72.5)	29 (82.86)	2 (40.0)	53 (51.96)	43 (25.0)	10 (27.03)
Use of next‐generation sequencing technologies, count (%)	96 (9.26)	58 (9.85)	12 (11.32)	7 (8.75)	3 (8.57)	2 (40.0)	3 (2.91)	16 (9.3)	2 (5.41)
Single‐center study, count (%)	522 (50.39)	344 (58.4)	19 (17.92)	39 (48.75)	7 (20.0)	1 (20.0)	42 (41.18)	71 (41.28)	14 (37.84)
Multicenter, same country, count (%)	230 (22.2)	117 (19.86)	32 (30.19)	32 (40.0)	15 (42.86)	1 (20.0)	25 (24.51)	18 (10.47)	9 (24.32)
Multicenter, international, count (%)	118 (11.39)	48 (8.15)	19 (17.92)	3 (3.75)	11 (31.43)	1 (20.0)	26 (25.49)	49 (28.49)	8 (21.62)
Collaborative network not clearly stated, count (%)	162 (15.64)	77 (13.07)	35 (33.02)	6 (7.5)	1 (2.86)	2 (40.0)	9 (8.82)	33 (19.19)	6 (16.22)

^a^ The same study may have included more than one ethnicity/region.

^b^ Defined as no more than 10 patients reported.

SD, standard deviation; PD, Parkinson's disease; URP, underrepresented population.

For data collection, the largest number of studies were conducted in a single‐center (522, 50.4%), 230 (22.2%) had multiple centers within the same country, and 118 (11.4%) had multiple international centers. Single‐center studies predominated in studies on participants of Chinese ancestry (58.4%), which presented a lesser proportion of international multicenter collaboration (8.2%). International multicenter studies were the lowest in South Asia (3.8%). A larger proportion of this type of study was observed in other regions, especially in Southeast Asia (31.4%) and the Middle East and North Africa (28.5%). Regarding the country of each coauthor, most studies included authors from the same country (71%). Studies with authors from multiple countries were lowest among South Asia (15.4%) and Greater China (23.7%) and highest in Central Asia (71.4%), the Middle East and North Africa (61.5%), and sub‐Saharan Africa and other Blacks and Southeast Asia (50%, each). In accordance with these results, the mean number of authors per publication was lowest in South Asia (7.1 ± 3.4) and Greater China (9.2 ± 6.8) and highest in Central Asia (16.9 ± 13.6).

**FIG 3:**
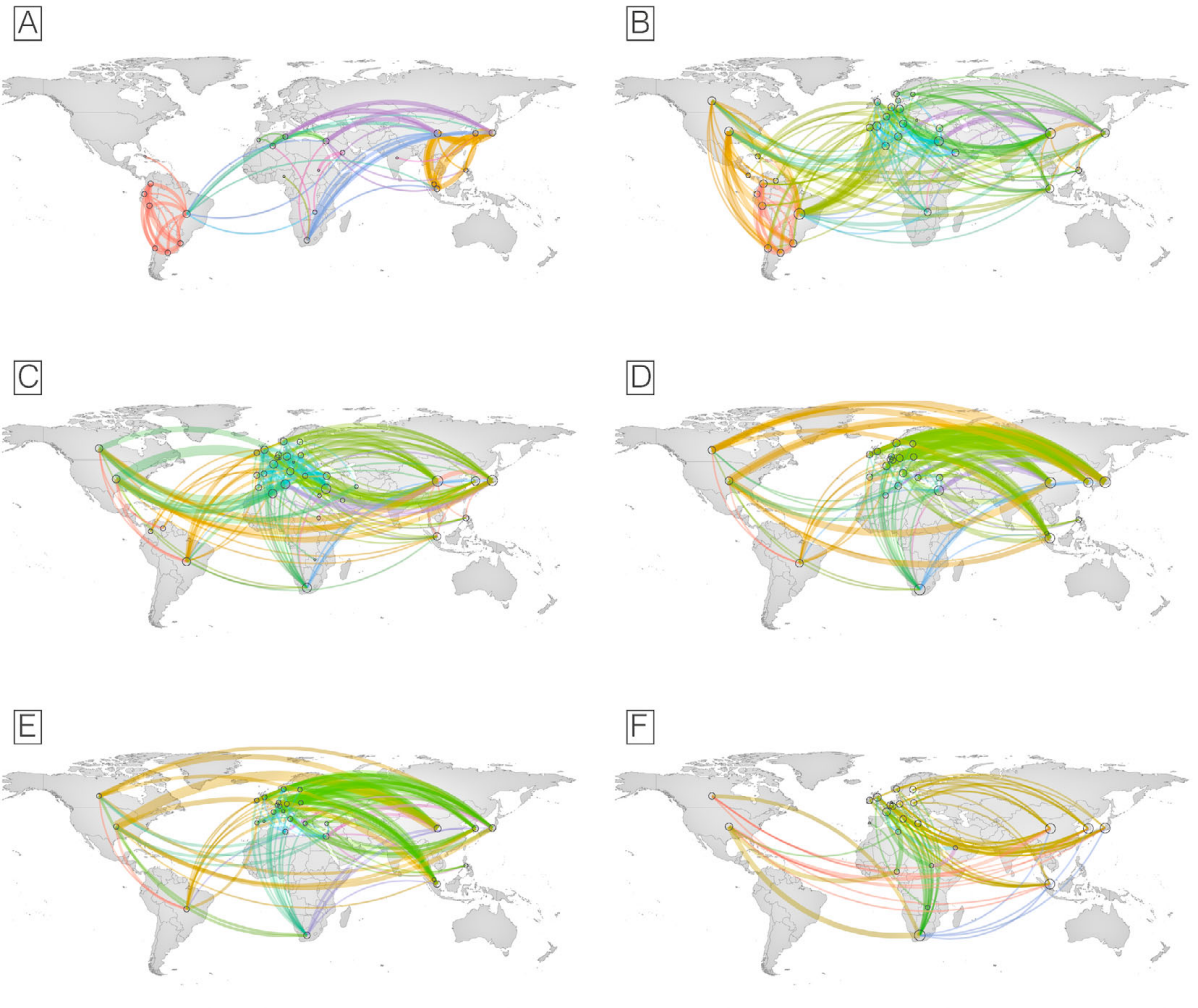
Country collaboration maps by studied populations. Panel A: Collaboration map excluding the Global North. Panel B: Collaboration map for articles describing Latin America & Caribbean populations. Panel C: Collaboration map for articles describing Middle East & North Africa populations. Panel D: Collaboration map for articles describing Asia excluding Greater China populations. Panel E: Collaboration map for articles describing Greater China populations. Panel E: Collaboration map for articles describing Greater China populations.

When examining the collaboration network maps (Fig. 3), we observe that sub‐Saharan African and Asian countries intensely collaborated with Europe, whereas Latin American and Caribbean countries collaborated equally with North America. Collaborations between countries with a high proportion of underrepresented groups were limited, mainly within the same region. Graph theory measures of the countries’ collaboration networks, including all publications, revealed similar classifications for degree centrality (DC) and closeness centrality (CC): USA (DC = 0.745, CC = 0.238), Germany (DC = 0.673, CC = 0.234), China (CC = 0.582, DC = 0.229), Canada (CC = 0.582, DC = 0.229), and the United Kingdom (CC = 0.582, DC = 0.229). The classification of betweenness centrality (BC) was slightly different: the United States (BC = 0.138), Germany (BC = 0.131), the United Kingdom (BC = 0.061), Australia (BC = 0.060), and France (BC = 0.041). For further comprehension, countries' collaboration networks, including publications involving each population, are presented in Supplementary Figure 2, along with graph theory measures for each network.

**FIG 4:**
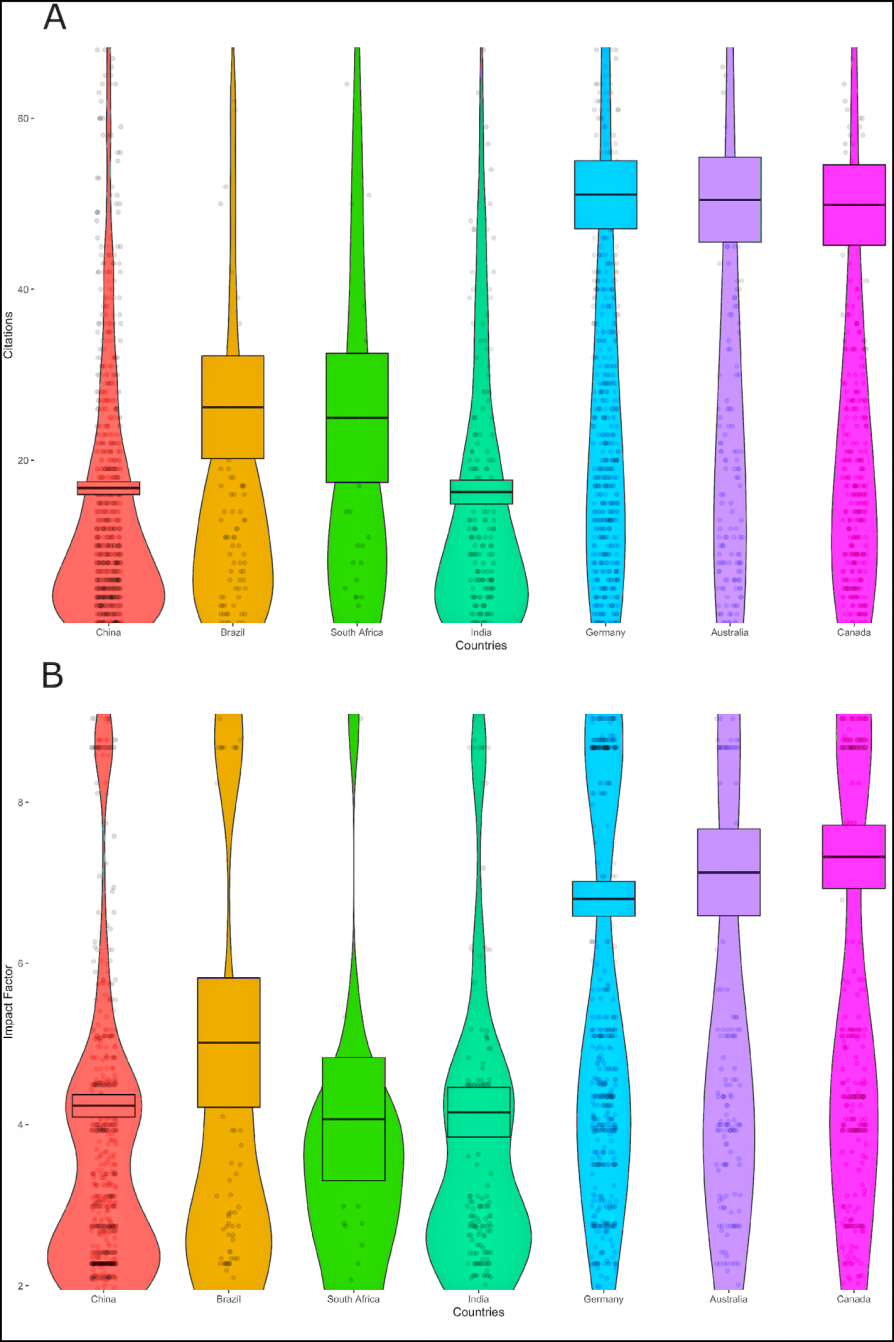
Citations and journal's impact factor across selected countries. Panel A: number of citations by country. Panel B: Journal's impact factor by country.

Southeast Asia presented the highest sample size of PD patients (median 386), followed by China (median 383). Latin America and Caribbean, sub‐Saharan Africa and other Blacks, and Central Asia had the lowest sample size (median: 138, 104, and 80, respectively). The sample size of controls followed the same pattern as that of patients. Case reports and case series (up to 10 patients included) represented 52 titles (5%), and the highest number was from the Middle East and North Africa (9.9%). The highest citations per document were those from Southeast Asia (5.2 ± 15.1) and Central Asia (4.8 ± 9.9), whereas East Asia, excluding Greater China (4.2 ± 11), and the Middle East and North Africa (4.1 ± 7.8) presented intermediate citations. The lowest citation count was among Greater China (1.8 ± 4.1), South Asia (1.5 ± 1.4), and sub‐Saharan Africa and other Blacks (1.8 ± 1.2). The average impact factor of the journals where the studies were published was highest in Southeast Asia (7.6 ± 13.4), the Middle East and North Africa (6.8 ± 10.1), and East Asia, excluding Greater China (6.1 ± 8.4), and lowest in South Asia (3.5 ± 2.0) and sub‐Saharan Africa and other Blacks (4.0 ± 2.2). In comparison to the three countries with a predominance of European ancestry, countries with a predominance of URPs publish in journals with a lower impact factor and obtain fewer citations (Fig. 4).

## Discussion

This review aimed to provide an overview of the current situation for PD genetics research among URPs, identify strengths and limitations, outline critical directions for future efforts, and set a baseline to measure their impact. We believe that the summary provided here represents a significant step forward to highlight disparities and foster representativeness, with the potential to prevent inequalities in the health care of PD patients. Notably, we observed considerable imbalances in PD genetic studies among URPs. Whereas Greater China was described in the majority of the articles published (57%), other ethnic groups were less well studied, for example, sub‐Saharan Africans and other Blacks representing just 4% of the publications. Although idiopathic PD was more studied than monogenic forms of the disease, most studies analyzed a limited number of genetic variants. We identified just nine studies using a genome‐wide approach published until 2021.

The bias towards European ancestry populations is a well‐established problem in genetics, especially in GWASs. Efforts have been initiated to address this by major research funders, but we are still far from the desired equity.^13^ To further understand this bias, our search focused on PD genetic studies performed in non‐European populations, which account for a great variety of ethnic backgrounds. Greater China populations are represented in more than half of the studies (57%), followed by the Middle East and North Africa, East Asia non‐Chinese, and Latin America and Caribbean. In addition, we observed that publication counts in Greater China are increasing annually, which is consistent with Popejoy and Fullerton,^1^ who stated that these groups were the most effective in their efforts to improve representation in genetic research.^1^ Besides having a vast population compared to the rest of the world, their economic growth with significant investments in science and education can explain this progress. For example, China increased more than 10% of its expenditure on science in 2020.^14^ On the contrary, Central Asia had just five papers published, all of them from researchers based in foreign countries. Black ancestry was represented in just 4% of the studies, without a perceptible trend of increase in the publication counts over the years. The majority of Blacks reside in sub‐Saharan Africa and Latin America, both regions with countries within the lower‐income and lower‐middle‐income strata. Consequently, economic constraints foster the limited expenditure on and development of research and innovation, in general, and for noncommunicable diseases such as PD, in particular. Another barrier in many parts of the region is the access to specialized health care, such as neurologists, to diagnose diseases. Finally, historical discrimination and misconceptions about the purpose of research also contribute to lower participation rates of this group in research studies.

The highest frequency of studies on idiopathic PD was in Greater China, with all the other populations showing a higher frequency of studies on monogenic forms of PD. They also presented the highest median sample size and a higher proportion of the studies funded exclusively by local resources. Studying a multifactorial disorder such as idiopathic PD is a logistical and financial challenge because large samples need to be recruited to have sufficient power to detect minor effects and control for confounders. Probably, studying monogenic forms is often a more straightforward endeavor for lower‐income countries because fewer participants are needed to be recruited. Genetic analysis for such rare forms, although expensive, can also usually be performed in partnership with laboratories from higher‐income countries. Following this observation and considering that the sample size can be an indirect indicator of study quality, we see that studies with Asian populations, especially Greater China, reported the largest sample size. However, regarding citations per publication and the journal's impact factor, another indirect index of quality, Greater China presented lower figures than other regions. Studies in the Greater China population have mainly investigated candidate genes in idiopathic PD, and to date, more comprehensive study designs like GWASs that can potentially generate more citations are still infrequent. Another potential explanation for this lower citation rate is that Greater China scientific publications have generally been more recent compared to the others. Southeast Asia is an exceptional case. Despite its still lower number of publications, it was able to recruit the largest sample sizes in individual studies, most of them locally funded, and exhibit the highest citations and impact factor. Countries with high economic development, such as Singapore and Malaysia, are in this region.

Collaborative studies are crucial in genetics when we need to gather a substantial sample size. Besides that, a research network can be beneficial for underserved countries because it can strengthen credibility, facilitate data sharing, and promote capacity building. Our collaboration network analysis showed that the main centrality measures indicate that developed countries such as the United States, Germany, and Canada play a significant role in promoting diversity through collaboration with countries with a predominance of URPs. Most of the samples collected for PD genetic studies in South Asian and Greater China populations were from single centers, and international collaborations were scarce. In South Asia and Southeast Asia, there were more local multicenter studies but still limited international collaborations. These observations are supported by other collaborative indicators, such as the number of authors per publication and the frequency of studies with authors from a single country, both lower among Asians. This trend might be partially explained by a higher research capacity in Asia, especially in East Asia and Southeast Asia. Stringent local regulations that govern data and biospecimen sharing, although intended to prioritize and develop local capacities, may also limit extensive international collaborations in several regions. A substantial obstacle to extensive partnerships could be a lack of trust between clinicians and researchers from varying socioeconomic backgrounds and academic evaluation systems. Investigators from less‐developed nations may be fearful about devoting significant effort to data collection that may go unrecognized in publications. Similarly, cooperation between researchers within nations with limited access to research funding may be concerned that sharing their data may benefit their “competitors.” Other factors that may further contribute to the limited collaborations might include language barriers and cultural issues discouraging collaborations. The highest indicators of collaboration were observed in the Middle East and North African studies, with more international multicenter studies and increased frequency of authors from multiple countries. A possible explanation for this is the higher frequency of *LRRK2* p.G2019S carriers in this region, which could have piqued international interest (due to its common occurrence in North Africa and Ashkenazi Jewish populations) and fostered international collaborations.^15^, ^16^ The need for high investment capital in cutting‐edge technologies might also promote collaborations with higher‐income countries.

A notable limitation of the present study can be the number of publications used as the primary measure of population representation. One could also argue that the broad search criteria used include such a diversity of studies that their joint numbers express vague concepts. Even so, we consider that our approach provides key indirect measures of scientific interest, development, and output in those specific populations, which reflect representation not only at the DNA level in databases but also at a much broader appraisal of the population's social, financial, and scientific aspects. It is also important to note that many studies relied on the same samples for their analyses, thus not adding diversity at the DNA level and increasing the chance of error by performing multiple comparisons. Furthermore, a thorough quality assessment of each study was not performed because we did not find an objective assessment tool, such as the CONSORT statement for clinical trials, which covers all the different study types included in our search. Also, this task would require extensive effort, which could prevent its replication in the future. Instead, we used indirect measures to estimate quality and also visibility, like citations and the journal's impact factor. Our aim was to assess the overall scientific production in PD genetics research, and we did not provide a summary of most frequent genes and mutations identified or detailed clinical characterization. However, we should mention that most of the papers failed to provide a clear description of phenotypic characteristics, not just in studies of sporadic cases but also in studies of suspected monogenic cases, for which phenotypes are even more critical. Finally, another limitation was the inclusion of only English‐language publications. Although possibly introducing selection bias for higher‐impact papers, this choice narrowed the analysis to articles with higher international visibility.

As pointed out by Popejoy and Fullerton,^1^ we believe future efforts to increase diversity in genomic research should include both bottom‐up and top‐down strategies. Researchers should acknowledge the importance of diversity in their studies, formulating questions and proposing robust study designs considering genomic diversity and its relationship with socioeconomic and environmental factors. Strategies to ensure recruitment among populations not used to participating in research include engaging local communities and proposing solutions to improve health care. In a heterogeneous condition such as PD, a thorough clinical characterization of participants is critical in ethnically diverse genetic studies. Efforts to increase genetic diversity must be coupled with efforts to standardize phenotypic descriptions and inclusion criteria to maximize the etiological implications of the research. From an analytical perspective, increasing information can be gained through tools like trans‐ethnic fine‐mapping.^17^ Furthermore, funding agencies should promote representation by providing dedicated funding, increasing diversity among researchers, and applying knowledge to health‐care systems.^13^

While foreseeing potentially vulnerable communities, stringent ethical procedures should guarantee participants' autonomy and dignity, including ethical oversight by culturally competent agents, a thorough informed consent process, respect for local regulations, data protection, and return of value.^18^, ^19^ The past exploitation and abuse of indigenous populations for genetic research has resulted in several emerging guidelines to protect these populations—this includes a code of ethics by the San people of southern Africa,^20^ recommendations issued by the Human Heredity and Health (H3Africa) Guidelines for Community Engagement,^21^ and a policy for genetic research and data sharing, being developed by the Navajo Nation in the United States.^22^ Establishing effective communication channels that enable collaborators to communicate their thoughts and concerns is critical for dissipating the lack of trust that can hinder partnerships. In addition, radical transparency should be implemented throughout all aspects of research, including goals, funding, governance, and publication policies. Capacity building in countries with a predominance of URPs is another crucial step to guarantee long‐term studies and build autonomy and a diverse pool of researchers with expertise in genetics research.^23^, ^24^, ^25^ Merely including the names of young investigators as coauthors in the middle of multiauthored articles is insufficient; a clear career development strategy for junior researchers should be implemented with a plan for them to produce first‐author or senior‐author publications in the future. As observed in our results, there is a surplus of opportunities for promoting sustainable diversity in genomics studies through the empowerment of local researchers and authorities. This could be achieved either through collaboration with higher‐income countries or by designing plans for regional development with specialized centers. International institutions have a key role in creating common fora for partnership development with higher‐income countries or organizing underserved countries for regional ventures. Moreover, journals and editors should be sensitive to the importance of increasing diversity in publications, and a first step would be to increase representativeness in editorial boards. Editions focused on URPs and specific criteria for publication could also be considered. Finally, peer review should be carefully conducted to be a supportive and productive process fostering diversity.

The Global Parkinson's Genetics Program (GP2, http://gp2.org/) is a project that aims to provide a comprehensive understanding of the genetic architecture of PD, utilizing strategies that include collecting large‐scale data from URPs worldwide and enabling researchers from those populations to drive this work forward.^26^ To accomplish this ambitious goal, GP2 established a specific working group (the Underrepresented Populations Working Group, https://www.gp2.org/working-groups/underrepresented-populations-working-group/) comprising researchers from different countries and ethnic backgrounds to ensure adequate global representation. For data collection, GP2 is creating a consortium and projects to recruit subjects from URPs inside the United States called the Black and African American Connections to Parkinson's Disease Study. Already existing initiatives in East Asia (IPDGC‐Asia), India (LUX‐Giant), Latin America (LARGE‐PD), and Africa (IPDGC‐Africa) receive strong support from the program. Besides data collection, strategies for collaborative data upload, access, and analysis are making it possible for GP2 to perform projects such as a trans‐ethnic meta‐analysis and fine‐mapping, involving a diversity of researchers, resources, and data. To foster collaboration and build resources, GP2 provides a wide range of training opportunities for researchers globally, including online courses, master's degrees and PhD programs, and short‐duration training visits on genetics and bioinformatics.

In conclusion, although steps have been taken globally to ensure diversity in PD genetic studies, the unbalanced efforts between URPs are still concerning, as highlighted here. Among growing economies, we observed a steady increase in publications over the years, whereas this rate has been slower in lower‐income regions. Concerted efforts are needed to recognize diversity as a driver of equality and scientific discoveries. Researchers, universities, and funders, either public or private, should assume more active roles in paving the paths to achieve sustainable diversity through joint efforts, capacity building, training, data sharing, and consciously redirecting capital. In this sense, GP2 is playing an ambitious role in unveiling PD's genetic architecture by engaging leaders, researchers, and study participants from Africa, Asia, Middle East, Latin America, and all other URPs within more developed nations. Hopefully, within the next few years, we will see a more inclusive research environment being translated into more higher‐quality publications among URPs, with potential parallel improvements in the health care of all populations.

### Relevant conflicts of interest/financial disclosures

The authors have no conflicts of interest to declare.

### Funding agency

This research was funded by Aligning Science Across Parkinson's through The Michael J. Fox Foundation for Parkinson's Research.

## Author Roles

- A.F.S.‐S.: design, execution, analysis, writing, and editing of the final version of the manuscript.
- A.B.: execution, analysis, writing, and editing of the final version of the manuscript.
- O.O.: design, execution, and editing of the final version of the manuscript.
- K.M.: design, execution, and editing of the final version of the manuscript.
- S.‐Y.L.: design, execution, and editing of the final version of the manuscript.
- S.B.: design, execution, and editing of the final version of the manuscript.
- A.A.A.: execution and editing of the final version of the manuscript.
- B.L.‐S.: execution and editing of the final version of the manuscript.
- M.Z.S.: execution and editing of the final version of the manuscript. M.S.: execution and editing of the final version of the manuscript.
- S.C.R.: execution and editing of the final version of the manuscript.
- Y.Z.Z.: execution and editing of the final version of the manuscript.
- S.D.: execution and editing of the final version of the manuscript.
- J.A.P.: execution and editing of the final version of the manuscript.
- L.K.P.: execution and editing of the final version of the manuscript.
- R.R.: execution and editing of the final version of the manuscript.
- A.J.N.: design, execution, and editing of the final version of the manuscript.
- N.O.: execution and editing of the final version of the manuscript.
- M.R.: design, execution, and editing of the final version of the manuscript.
- S.L.: design, execution, and editing of the final version of the manuscript.
- I.M.: design, execution, and editing of final version of the manuscript.

## Full financial disclosures of all authors for the previous 12 months

- A.F.S.‐S.: grants: ASAP‐GP2, The Michael J. Fox Foundation, Fundação de Amparo à Pesquisa do estado do RS (Fapergs, Brazil) and Conselho Nacional de Desenvolvimento Científico e Tecnológico (CNPq, Brazil).
- A.B.: Coordenação de Aperfeiçoamento de Pessoal de Nível Superior 88882.345554/2019‐01.
- O.O.: none.
- K.M.: none.
- S.‐Y.L.: consultancies: The Michael J. Fox Foundation, Lundbeck International Neuroscience Foundation Editorial Board; honoraria for lecturing: International Parkinson and Movement Disorder Society (MDS), International Brain Research Organization, Lundbeck, and Medtronic; and grants: The Michael J. Fox Foundation and the Malaysian Ministry of Education Fundamental Research Grant Scheme.
- S.B.: South African Medical Research Council (Self‐Initiated Research Grant), the National Research Foundation of South Africa (grant number: 129249), and the South African Medical Research Council/Stellenbosch University Genomics of Brain Disorders Research Unit.
- A.A.A.: grants: Malaysian Ministry of Higher Education, The Michael J. Fox Foundation, and ALS Association.
- B.L.‐S.: none.
- M.Z.S.: none.
- M.S.: grants: Bartlett Fund for Critical Challenges, The American University in Cairo, German Academic Exchange Services (DAAD), Technical University of Munich Global Incentive Fund, and the Egyptian Academy of Scientific Research and Technology (ASRT); and honoraria: Movement Disorders Society.
- S.C.R.: none.
- Y.Z.Z.: none.
- S.D.: none.
- J.A.: none.
- L.K.P.: consultancies: Medgenonme Labs Pvt Ltd; and honoraria: Sun Pharmaceuticals, Cipla, and Intas Pharmaceuticals.
- R.R.: grants: DBT India; All India Institute of Medical Sciences, New Delhi; and The Michael J. Fox Foundation.
- A.J.N.: grants: Parkinson's UK, Barts Charity, Cure Parkinson's, Virginia Keiley Benefaction, Alchemab, Aligning Science Across Parkinson's, and The Michael J. Fox Foundation; and consultancy and personal fees: Astra Zeneca, AbbVie, Profile, Roche, Biogen, UCB, Bial, Charco Neurotech, uMedeor, and Britannia.
- N.O.: grants: The Michael J. Fox Foundation; and honoraria: International Parkinson and Movement Disorders Society.
- M.R.: grants: The Michael J. Fox Foundation, UCL Grand Challenges, and UCL Global Engagement.
- S.L.: grants: Fondation pour la Recherche Médicale (MND202004011718).
- I.M.: grants: NIH, The Michael J. Fox Foundation, ASAP‐GP2, and Cleveland Clinic Foundation.

## Supporting information


**APPENDIX S1.** Supporting Information

**Supplementary Figure 1:** Flowchart of the systematic review process

**Supplementary Figure 2:** Countries collaboration networks. Networks of scientific collaboration among the first authors' countries of our retrieved bibliographic data using (A) all the data, or only publications that included population data from (B) Sub‐Saharan Africans of Other Blacks, (C) Latin Americans, (D) Asians, and (E) Middle Easterns and North Africans. Boxes present the main graph measures. Clustering was performed using the Louvain method.

**Supplementary Table 1:** Detailed search string.

**Supplementary Table 2:** Complete list of the references included.

**Supplementary Table 3:** Molecular biology techniques used in studies of familial/monogenic forms of PD.

**Supplementary Table 4:** List of GWAS studies included.

**Supplementary Table 5** Molecular biology techniques used in the studies.

## Data Availability

Raw and derived data supporting the findings of this study are available from the corresponding author on request.
