# Supplementary figures and images for: Underrepresented Populations in Parkinson's Genetics Research: Current Landscape and Future Directions
Source: Mov Disord. 2022 Jul 22;37(8):1593–604. doi: 10.1002/mds.29126 (PMC10360137; doi:10.1002/mds.29126)

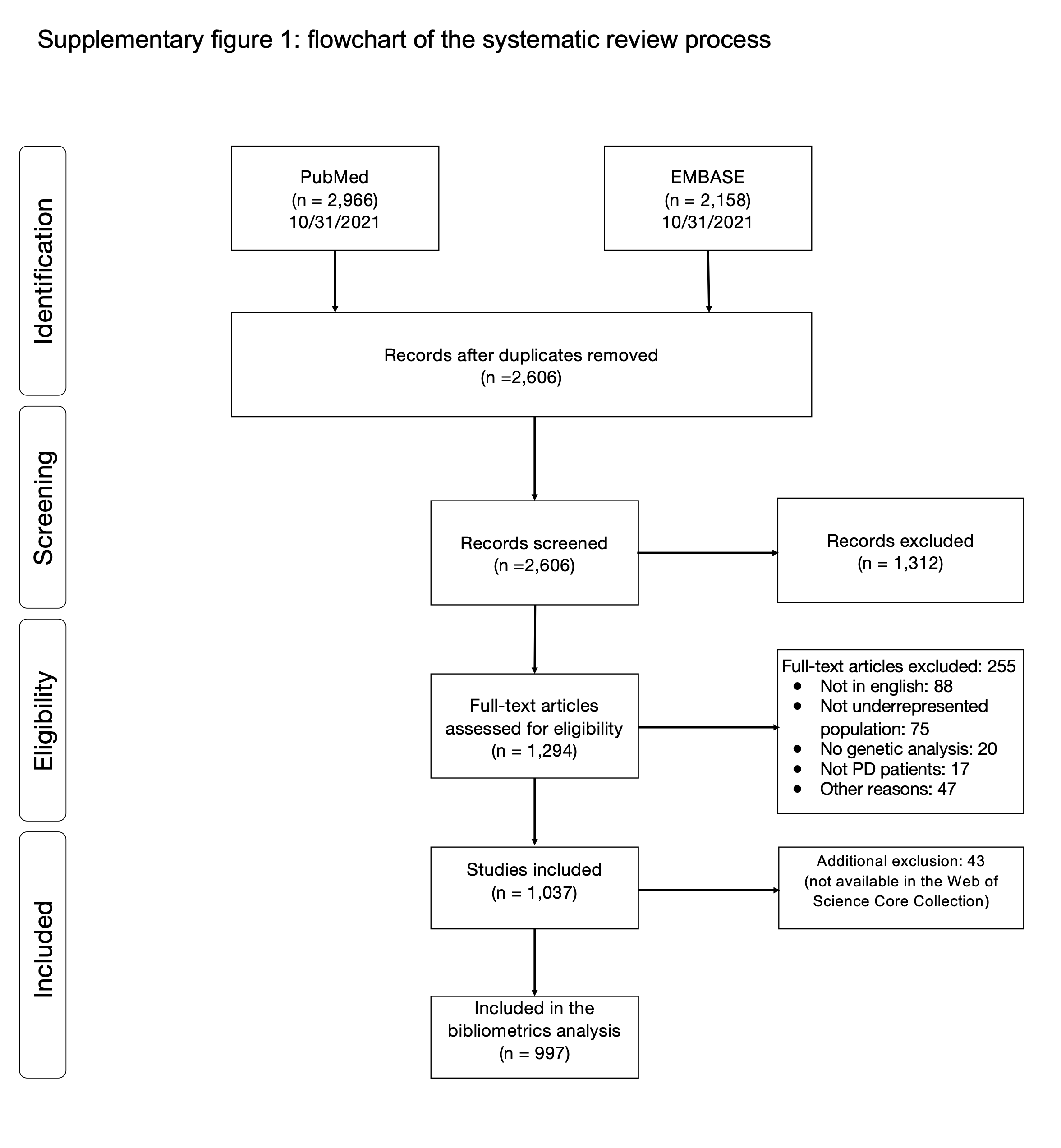

Supplement: Supplementary file 2 — Supplementary Figure 1: Flowchart of the systematic review process [file MDS-37-1593-s004.tiff]
