# Supplementary material for: Underrepresented Populations in Parkinson's Genetics Research: Current Landscape and Future Directions
Source: Mov Disord. 2022 Jul 22;37(8):1593–604. doi: 10.1002/mds.29126 (PMC10360137; doi:10.1002/mds.29126)
Supplement: Supplementary file 6 — Supplementary Table 3: Molecular biology techniques used in studies of familial/monogenic forms of PD. [file MDS-37-1593-s007.docx]

Supplementary Table 3: Molecular biology techniques used in studies of familial/monogenic forms of PD*

|  | **All studies** | **Greater China** | **East Asian (other than Greater China)** | **South Asia** | **Southeast Asia** | **Central Asia** | **Latin American & Caribbean** | **Middle Eastern & North Africa** | **Sub Sahara African or other Blacks** |
| --- | --- | --- | --- | --- | --- | --- | --- | --- | --- |
| Single gene study, count, (%) | 265 (75.28) | 92 (75.41) | 40 (88.89) | 34 (80.95) | 8 (72.73) | 3 (100.0) | 38 (71.7) | 69 (72.63) | 18 (62.07) |
| Multiple gene study, count, (%) | 87 (24.79) | 30 (24.59) | 5 (11.11) | 8 (19.05) | 3 (27.27) | 0 (0.0) | 15 (28.85) | 26 (27.37) | 11 (37.93) |
| Conventional sequencing (Sanger), count, (%) | 219 (62.22) | 77 (63.11) | 35 (77.78) | 27 (64.29) | 8 (72.73) | 1 (33.33) | 41 (77.36) | 54 (56.84) | 16 (55.17) |
| Next-generation targeted sequencing, count, (%) | 26 (7.39) | 12 (9.84) | 5 (11.11) | 2 (4.76) | 2 (18.18) | 0 (0.0) | 1 (1.89) | 3 (3.16) | 1 (3.45) |
| Whole-exome sequencing, count, (%) | 33 (9.38) | 23 (18.85) | 2 (4.44) | 3 (7.14) | 0 (0.0) | 1 (33.33) | 1 (1.89) | 6 (6.32) | 0 (0.0) |
| Whole-genome sequencing, count, (%) | 4 (1.14) | 2 (1.64) | 1 (2.22) | 1 (2.38) | 0 (0.0) | 0 (0.0) | 0 (0.0) | 0 (0.0) | 0 (0.0) |
| Genotyping by PCR products followed by electrophoresis**, count, (%) | 53 (15.06) | 20 (16.39) | 5 (11.11) | 6 (14.29) | 0 (0.0) | 0 (0.0) | 12 (22.64) | 9 (9.47) | 5 (17.24) |
| Real-time PCR genotyping (Taqman assays), count, (%) | 56 (15.91) | 16 (13.11) | 9 (20.0) | 4 (9.52) | 1 (9.09) | 0 (0.0) | 13 (24.53) | 13 (13.68) | 4 (13.79) |
| Mass spectrometer genotyping, count, (%) | 10 (2.84) | 4 (3.28) | 0 (0.0) | 2 (4.76) | 0 (0.0) | 0 (0.0) | 1 (1.89) | 4 (4.21) | 1 (3.45) |
| High-throughput genotyping, count, (%) | 10 (2.84) | 1 (0.82) | 1 (2.22) | 2 (4.76) | 0 (0.0) | 0 (0.0) | 1 (1.89) | 4 (4.21) | 1 (3.45) |

The same study may have included more than one molecular biology technique.

*Defined as studies including subjects with a clear family history of PD or reporting results in known causative mutations in PD genes.

**Including restriction fragment length polymorphisms.
