# Supplementary material for: Underrepresented Populations in Parkinson's Genetics Research: Current Landscape and Future Directions
Source: Mov Disord. 2022 Jul 22;37(8):1593–604. doi: 10.1002/mds.29126 (PMC10360137; doi:10.1002/mds.29126)
Supplement: Supplementary file 8 — Supplementary Table 5 Molecular biology techniques used in the studies. [file MDS-37-1593-s006.docx]

Supplementary Table 5: Molecular biology techniques used in the studies

|  | **All studies** | **Greater China** | **East Asian (other than Greater China)** | **South Asia** | **Southeast Asia** | **Central Asia** | **Latin American & Caribbean** | **Middle Eastern & North Africa** | **Sub Sahara African or other Blacks** |
| --- | --- | --- | --- | --- | --- | --- | --- | --- | --- |
| Count (%) | 1037 | 589 | 107 | 80 | 38 | 5 | 103 | 172 | 37 |
| Conventional sequencing (Sanger), count, (%) | 419 (40.41) | 222 (37.69) | 49 (45.79) | 38 (47.5) | 15 (39.47) | 1 (20.0) | 51 (49.51) | 75 (43.6) | 20 (54.05) |
| Next-generation targeted sequencing, count, (%) | 38 (3.66) | 19 (3.23) | 8 (7.48) | 2 (2.5) | 3 (7.89) | 1 (20.0) | 1 (0.97) | 5 (2.91) | 1 (2.7) |
| Whole-exome sequencing , count, (%) | 51 (4.92) | 37 (6.28) | 4 (3.74) | 4 (5.0) | 0 (0.0) | 1 (20.0) | 2 (1.94) | 8 (4.65) | 1 (2.7) |
| Whole-genome sequencing, count, (%) | 11 (1.06) | 6 (1.02) | 1 (0.93) | 1 (1.25) | 0 (0.0) | 0 (0.0) | 0 (0.0) | 3 (1.74) | 0 (0.0) |
| Genotyping by PCR products followed by electrophoresis*, count, (%) | 281 (27.1) | 157 (26.66) | 23 (21.5) | 28 (35.0) | 5 (13.16) | 0 (0.0) | 31 (30.1) | 41 (23.84) | 6 (16.22) |
| Real-time PCR genotyping (Taqman assays), count, (%) | 210 (20.25) | 96 (16.3) | 27 (25.23) | 14 (17.5) | 13 (34.21) | 1 (20.0) | 33 (32.04) | 39 (22.67) | 9 (24.32) |
| Mass spectrometer genotyping, count, (%) | 76 (7.33) | 65 (11.04) | 3 (2.8) | 2 (2.5) | 1 (2.63) | 0 (0.0) | 1 (0.97) | 8 (4.65) | 1 (2.7) |
| High-throughput genotyping, count, (%) | 22 (2.12) | 7 (1.19) | 4 (3.74) | 2 (2.5) | 0 (0.0) | 0 (0.0) | 2 (1.94) | 7 (4.07) | 1 (2.7) |
| SNP microarray, count, (%) | 130 (12.54) | 90 (15.28) | 12 (11.21) | 4 (5.0) | 1 (2.63) | 0 (0.0) | 8 (7.77) | 22 (12.79) | 2 (5.41) |

The same study may have included more than one molecular biology technique.

*Including restriction fragment length polymorphisms.
